# Acute Disseminated Encephalomyelitis Following Thoracic Endovascular Aortic Repair

**DOI:** 10.7759/cureus.30562

**Published:** 2022-10-21

**Authors:** Cleofina Furtado, Changrez Jadun, Sachin Srivastava, Sanjeev Nayak, Zafar Hashim

**Affiliations:** 1 Department of Diagnostic and Interventional Radiology, University Hospitals of North Midlands NHS Trust, Stoke-on-Trent, GBR

**Keywords:** demyelination, magnetic resonance imaging (mri), computed tomography (ct), thoracic endovascular aortic repair (tevar), acute disseminated encephalomyelitis (adem)

## Abstract

Acute disseminated encephalomyelitis (ADEM) is a central nervous system demyelinating condition. The postulated etiology is an autoimmune reaction, however, the mechanistic details are yet unknown. While infection and immunization are the most prevalent precipitating causes of ADEM, we postulate that radiopaque contrast material might have played a role as an immunological trigger. This presentation is unusual and has not been recorded so far. This case also emphasizes the challenges involved in evaluating emerging neurological problems following a period of intubation/sedation among these patients.

We present a patient who passed away due to the manifestations of ADEM following admission to the intensive care unit after thoracic endovascular aortic repair for type B aortic dissection. Magnetic resonance imaging (MRI) performed following multiple attempts at sedation weaning demonstrated typical features of this condition.

Clinicians and radiologists should be diligent and consider ADEM as a differential diagnosis when treating patients who present with neurological symptoms following radiological interventional procedures and have a high index suspicion to reduce mortality and achieve satisfactory clinical outcomes.

## Introduction

Acute disseminated encephalomyelitis (ADEM) has been well-known as a virus or vaccine-induced monophasic inflammatory myelinating autoimmune reaction against myelin [1]. ADEM often occurs relatively late after the beginning of the infection or trigger factor and is distinguished by increased white matter damage [2]. Encephalopathy with rapid neurologic impairments and brain magnetic resonance imaging (MRI) findings consistent with multifocal demyelination describe this condition [3]. It is more common in children and young adults, while occurrence in middle age or elderly individuals is uncommon [4,5].

SARS-CoV-2, varicella-zoster, Measles, rubella, mumps, Epstein-Barr virus, Coxsackievirus, cytomegalovirus, hepatitis A, and herpes simplex virus are all viral diseases linked to ADEM [6-8]. Mycoplasma pneumonia is the most common, with Leptospira, *Borrelia burgdorferi*, and group-A beta-hemolytic streptococci being the other bacterial illness [7,8]. Anti-rabies, pertussis, diphtheria, measles, mumps, rubella, and influenza immunizations are also linked to ADEM with growing cases also noted post-coronavirus disease 2019 (COVID-19) vaccination in the recent era [8,9].

We present this unique case of ADEM which occurred following thoracic endovascular aortic repair. Because of its unique occurrence, presentation of the disease, and prognosis; it was found worthy to be mentioned.

## Case presentation

**Figure 1:**
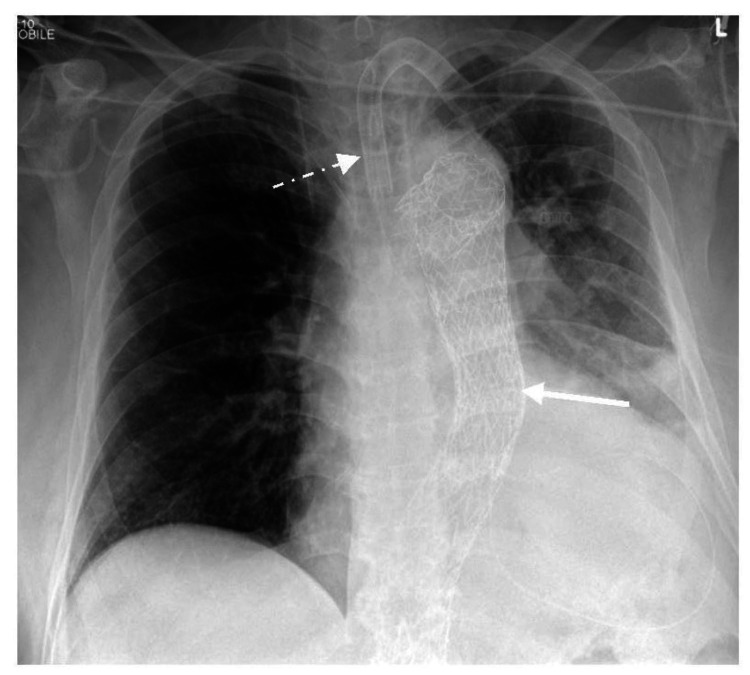
Supine anteroposterior (AP) chest radiograph. Bilateral lungs do not demonstrate any abnormal imaging features. The image shows post-TEVAR metallic stent (white solid arrow) and endotracheal tube (white dashed arrow). TEVAR: thoracic endovascular aortic repair

A 62-year-old patient was hospitalized in the intensive care unit following an elective thoracic endovascular aortic repair (TEVAR) of type B aortic dissection for post-procedural care. He was intubated and ventilated three days after due to worsening breathing and requirement for oxygen with a respiratory rate of 43, heart rate of 138 beats per minute, and blood pressure of 180/80 mmHg with normal temperature. The cause of these symptoms was unclear at that time. The chest radiographs (Figure 1) and computed tomography (CT) of the chest did not reveal any untoward with unremarkable appearance of the TEVAR graft and absence of pulmonary embolism.

There was no history of immunization, infection, or vaccination in recent days. He had otherwise healthy with no known chronic illness or drug use prior to the procedure. Polymerase chain reaction was negative for all major bacterial, viral, or fungal etiologies and cultures did not display any growth. Further blood cultures were negative for toxoplasmosis, HIV, hepatitis, and tuberculosis. Laboratory results revealed mildly elevated c-reactive protein 38 mg/L (0-5 mg/L), however, white cell count was normal. All other parameters were within a normal range including hemoglobin (Hb), liver and renal function tests, electrolytes, bone profile, and urinalysis.

Multiple trials were made to wean off sedation. On day 11, sedation was reduced significantly at which point he had neurological evaluation. He had a right gaze preference while preserving a normal doll's eye reflex. There was no vocal response to pain, and the only motor response was right-hand twitching. He was hypotonic throughout and had no bilateral limb responses.

**Figure 2:**
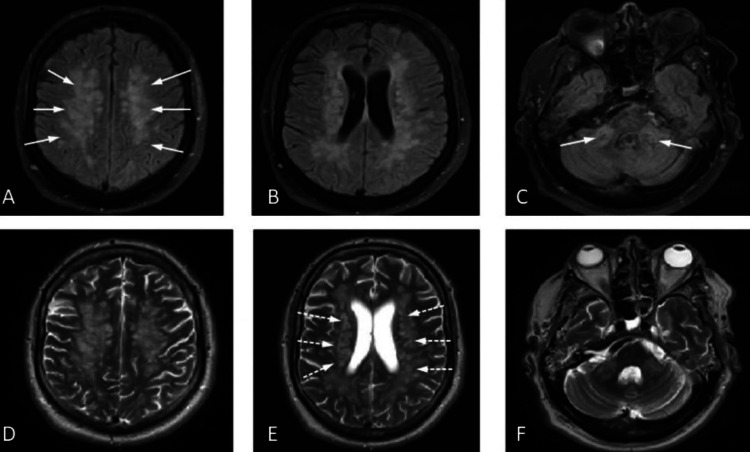
MRI brain axial FLAIR (A-C) and axial T2WI (D-F) demonstrate periventricular white matter high signal intensities on FLAIR (white solid arrows) and T2WI (white dashed arrows). FLAIR: fluid-attenuated inversion recovery; T2WI: T2-weighted imaging

**Figure 3:**
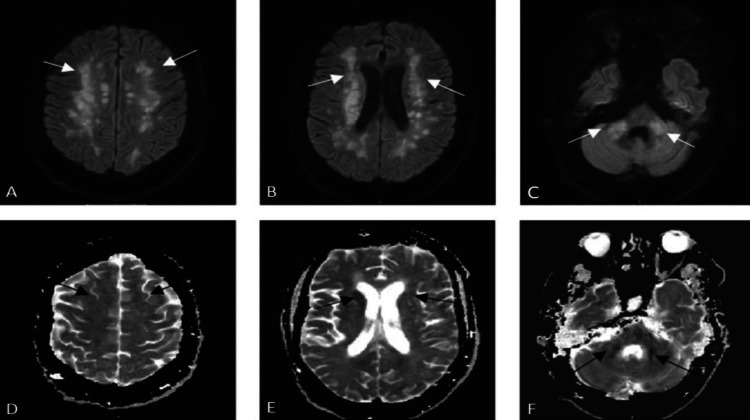
MRI brain axial DWI (A-C) and ADC (D-F) demonstrate abnormal diffusion restriction with high signal on DWI (white arrows) and low signal on ADC map (black arrows) in the corresponding white matter lesion. DWI: diffusion-weighted imaging; ADC: apparent diffusion coefficient

**Figure 4:**
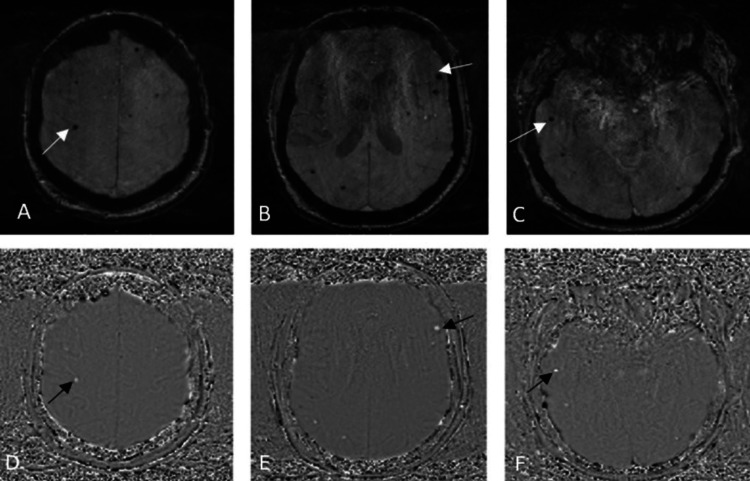
MRI brain axial SWI (A-C) demonstrates tiny areas of blooming artifacts (white arrows). Corresponding phase imaging (D-F) shows high signal (black arrows) keeping with hemosiderin deposition. SWI: susceptibility weighted imaging

Magnetic resonance imaging (MRI) was performed to look for a cause of persistent low Glasgow Coma Score (GCS) and hypotonia following the sedation wean which showed multiple patchy confluent T2 weighted imaging (T2WI) and fluid-attenuated inversion recovery (FLAIR) hyperintense lesions involving cerebral white matter (callosal, pericallosal, deep periventricular, basal ganglia), brainstem, and bilateral cerebellar peduncles (Figures 2A-2F). These lesions demonstrated diffusion restriction with some showing avid contrast enhancement (Figures 3A-3F). No pathological leptomeningeal enhancement was demonstrated after gadolinium contrast. Susceptibility weighted imaging (SWI) sequence demonstrated multiple foci of hemosiderin deposition in the cerebral cortices bilaterally, thalamus, and cerebellar hemispheres (Figures 4A-4F). The imaging findings were characteristic of ADEM. There was no evidence of aneurysm or venous thrombosis.

The electroencephalogram (EEG) demonstrated generalized slowing of sleep activity. Cerebrospinal fluid analysis (CSF) following lumbar puncture revealed lymphocytic pleocytosis, raised protein, normal glucose, and the absence of oligoclonal bands supporting the diagnosis ADEM over multiple sclerosis. CSF cytology showed no immunophenotypic evidence of a clonal B- or T-cell population with unremarkable bacterial culture, viral and meningoencephalitis PCR panel.

On funduscopic inspection, both optical discs were unremarkable. Serum was negative for anti-dsDNA, antinuclear antibody (ANA), rheumatoid factor, Sjögren syndrome type A (SSA), Sjögren syndrome type B (SSB), Smith/ribonucleoprotein (SM/RNP), scleroderma (SCL-70), myeloperoxidase, myelin oligodendrocyte glycoprotein, and aquaporin 4 receptor antibodies. The blood was also negative for varicella, cytomegalovirus (CMV), herpes simplex virus (HSV), rubella, syphilis, Borrelia, and Brucella. Tumor markers screening for cancer were negative. The laboratory studies also ruled out any toxic causes.

The patient showed mild neurological improvement with visual follow-up following pulse of 1000 mg of methylprednisolone treatment for five consecutive days. A total of five cycles of plasmapheresis every other day was administered with no significant improvement. One month following hospitalization he also developed multiorgan failure with anuria requiring continuous hemodiafiltration. Over the next few days, he remained critically unwell with a high level of inflammatory markers and no signs of neurological improvement. Following a discussion with his family, it was decided to withdraw support following which he passed away.

## Discussion

TEVAR has enabled a less invasive method to treat a variety of thoracic aortic diseases and is generally performed in surgically repaired type A dissection and those with a complicated type B dissection [10]. ADEM after TEVAR has not yet been documented. We postulate that the radiopaque contrast medium might have triggered an immune-mediated response, resulting in ADEM. The neurotoxic effects of iodinated contrast agents after coronary angiography reported so far include cortical blindness, altered mental status, seizures, spinal myoclonus, limb paralysis and weakening, and, in rare cases, coma [11]. Acute delirium and long-term cognitive impairment are some of the most commonly documented post-operative neurocognitive disorders after surgery. These are known to develop following regional or general anesthesia [12]. This, in conjunction with surgical trauma, causes cellular damage, stimulating endogenous factors that activate immune cells such as neutrophils and monocytes to resolve the damage and restore homeostasis. Activation of these cells contributes to neuroinflammation [13].

The exact mechanism of ADEM is not completely understood, but it is thought to result from inflammation triggered by an environmental stimulus or a foreign antigen that causes vascular congestion and increased permeability of the vasculature of the central nervous system [14]. This is expected to set off an inflammatory cascade including edema, inflammatory cell infiltration and perivenous hemorrhage leading to gliosis, demyelination, and necrosis [15].

Neurologic symptoms are variable, with the majority exhibiting encephalopathy, which includes confusion and lethargy, with difficulty awakening from sedation as the most common presentation [16]. These characteristics have the potential to hide an underlying inflammatory central nervous system condition in the critical care setting, where clinical neurological assessment is particularly difficult [6]. In addition, para-infectious neuropathological processes often manifest after a latent time following an infection, raising the clinical challenge of when to evaluate emerging neurological problems following a period of intubation along with challenges involved in transporting the patient for more complex imaging studies [17].

Histology is characterized by tiny (5 mm) white matter lesions with macrophage clusters and a variety of related axonal damage [17]. Acute hemorrhagic leukoencephalopathy has more severe histology than ADEM, with an enlarged swollen brain and punctate hemorrhages accompanied by fibrinoid necrosis in blood vessels and perivascular infiltrates containing neutrophils and eosinophils [18].

MRI shows lesions with a high sensitivity and may help rule out other entities that look similar, such as multiple sclerosis, which displays lesions around the deep white matter, near the corpus callosum or callososeptal junction [19]. FLAIR hyperintensities in deep white matter and at the grey/white matter interface with diffusion restriction are typical MRI lesions. Punctate or rim-enhancing lesions are seen on post-contrast enhancement [20].

## Conclusions

While infection and vaccination are the most common associations for ADEM, we suspect that radiopaque contrast material might have been a possible immunological trigger element in this case. The patient's clinical course demonstrates that during lengthy hospitalizations, neurological manifestations of ADEM might arise which are especially difficult to assess and recognize in critically unwell patients. High index suspicion and a low threshold for performing MRI are required in patients with neurological symptoms for an early diagnosis that will enhance prognosis.
